# A novel *de novo* variant of *GABRA1* causes increased sensitivity for GABA *in vitro*

**DOI:** 10.1038/s41598-020-59323-6

**Published:** 2020-02-11

**Authors:** Friederike Steudle, Sabah Rehman, Konstantina Bampali, Xenia Simeone, Zsofia Rona, Erwin Hauser, Wolfgang M. Schmidt, Petra Scholze, Margot Ernst

**Affiliations:** 1 Department of Pathobiology of the Nervous System, Center for Brain Research, Medical University Vienna, Vienna, Austria; 2 Department of Molecular Neurosciences, Center for Brain Research, Medical University Vienna, Vienna, Austria; 3 Landesklinikum Thermenregion Mödling, Department of Pediatrics, Mödling, Austria; 4 Neuromuscular Research Department, Center for Anatomy and Cell Biology, Medical University of Vienna, Vienna, Austria

**Keywords:** Neurodevelopmental disorders, Ion channels in the nervous system

## Abstract

The *GABRA1* gene encodes one of the most conserved and highly expressed subunits of the GABA_A_ receptor family. Variants in this gene are causatively implicated in different forms of epilepsy and also more severe epilepsy-related neurodevelopmental syndromes. Here we study functional consequences of a novel *de novo* missense *GABRA1* variant, p.(Ala332Val), identified through exome sequencing in an individual affected by early-onset syndromic epileptic encephalopathy. The variant is localised within the transmembrane domain helix 3 (TM3) and *in silico* prediction algorithms suggested this variant to be likely pathogenic. *In vitro* assessment revealed unchanged protein levels, regular assembly and forward trafficking to the cell surface. On the functional level a significant left shift of the apparent GABA potency in two-electrode voltage clamp electrophysiology experiments was observed, as well as changes in the extent of desensitization. Additionally, apparent diazepam potency was left shifted in radioligand displacement assays. During prenatal development mainly alpha2/3 subunits are expressed, whereas after birth a switch to alpha1 occurs. The expression of alpha1 in humans is upregulated during the first years. Thus, the molecular change of function reported here supports pathogenicity and could explain early-onset of seizures in the affected individual.

## Introduction

Gamma-aminobutyric acid (GABA) is a secreted transmitter that transduces signals from cell to cell by binding to membrane bound GABA receptors. Binding of GABA to GABA_A_ receptors opens an ion channel, allowing chloride or hydrogencarbonate ions to pass and thereby changing the membrane potential. While playing many roles in a wide range of tissues, the family of GABA_A_ receptors is most studied as central nervous system mediator of fast GABA-ergic neurotransmission^1,2,3^.

GABA_A_ receptors are either homopentamers or heteropentamers composed of up to four different subunits. Heteropentamers are assembled from a pool of 19 different subunits producing a large variety of receptor subtypes^1,4–6^.

The GABA system is involved in embryonic neurodevelopment^7–9^. In rodents, the GABA system can be detected as early as E12 and evolves before the glutamatergic system^9^. GABA has multiple functions during neurodevelopment, such as regulating neuronal cell proliferation, maturation and migration^8,9^. It exerts excitatory responses as well as trophic effects during prenatal development serving essential functions during neurogenesis^2^. During birth, maternal oxytocin triggers a switch in GABAergic function from excitatory to inhibitory action in the newborn^10^. This is accompanied by a switch in subunit expression. The 19 subunits differ in their developmental spatio-temporal expression. Different cell types express very specific GABA_A_ receptor genes during defined time-points of development. The main GABA_A_ receptor population in the neonatal brain is thought to contain two α2/3 subunits together with two β2/3 subunits and a γ2 subunit. The expression of α1 is very low in pre- and perinatal rodent brain, but it increases dramatically after birth in all brain regions^6,11–13^. In the adult mammalian brain, α1 contributes to the majority of GABA_A_ receptors^11^.

In humans, the upregulation of *GABRA1* expression after birth was shown to occur over the first postnatal years^14,15^. It is highly expressed during adulthood in mammalian species and contributes to multiple different receptor subtypes^1,11,14,16^. These receptor subtypes include synaptic ones, such as the highly expressed benzodiazepine sensitive α1βxγ2 GABA_A_ receptors as well as extrasynaptic ones like α1βx binary and α1βxδ receptors. GABA_A_ receptors are members of the superfamily of Cys-loop receptors. Structurally, each subunit consists of three domains: the extracellular domain (ECD) is located at the N-terminal end. This domain is followed by four transmembrane segments (helices TM1-4), forming a four helix bundle, the transmembrane domain (TMD), of which the TM2 helices form the ion pore. An intracellular domain (ICD, sometimes called “loop”) connects TM3 and TM4. Multiple ligand binding pockets exist within subunits and at the interfaces between neighboring subunits. In these pockets, different types of ortho- and allosteric ligands, including the agonist GABA, and modulators such as neurosteroids can bind^17^. For α1, the ECD contributes to the GABA binding site at the complementary face and to the benzodiazepine binding site at the principal face if the complementary neighbour is a γ subunit.

Genetic variants in genes encoding for GABA_A_ receptor subunits are widespread. Many of them seem to be naturally occurring polymorphisms, which do not cause any specific phenotype. Other variants are disease associated and are widely studied in brain-related disorders. This includes pathogenic *GABRA1* variants causing susceptibility to idiopathic generalized epilepsy (EIG13, OMIM 611136) or more severe epilepsy-related neurodevelopmental syndromes (e.g. early infantile epileptic encephalopathy, EIEE19; OMIM 615744)^18^.

The first link between a *GABRA1* variant and epilepsy was reported in 2002 as missense variant, NM_000806.5:c.965C > A p.(Ala322Asp), causing an amino acid exchange of a highly conserved alanine residue to aspartate in TM3 (A322D)^19,20^. In 2006 another variant within TM3 was identified: a frame-shift causing single-base deletion in *GABRA1* (NM_000806.5:c.975delC p.(Ser326Glnfs*3)) associated with childhood absence epilepsy^20,21^. Since then, more than 30 different variants, the vast majority of which are missense variants, have been identified in association with different forms of epilepsy or epilepsy-related syndromes^22^.

*In vitro* experiments indicate that many GABA_A_ receptors containing mutated α1 subunits often exhibit a lower sensitivity for GABA and reduced current amplitudes. This suggests a decrease in inhibitory activity and therefore increased susceptibility to seizures *in vivo*^22^. The reduction of GABA evoked currents can be due to different mechanisms, such as improper folding (A322D in TM3 inhibits transmembrane helix formation), changed receptor trafficking leading to retention in the endoplasmic reticulum (ER) and ER associated degradation (caused by the frame-shift S326fs328*) or nonsense mediated mRNA decay^20,21,23^.

Here, we report the identification and *in vitro* characterization of a novel *de novo* variant in an individual affected by a severe early-onset epilepsy-related neurodevelopmental syndrome.

## Results

### Clinical data and genetic analysis

The affected individual, a 13 year old boy, was initially diagnosed with early-onset refractory seizures, and neurodevelopmental retardation. Currently, he has muscle hypotonia and severe psychomotor impairment. His facial features are dysmorphic (with brachycephaly and microcephaly). He needs percutaneous endoscopic gastrostomy (PEG) feeding since the age of 5 years.

The affected individual was born at 39 + 3 weeks of gestation (spontaneous delivery) to healthy parents of Chechen descent with unremarkable family history. The first epileptic seizure was observed at the age of 2 ½ months. At the age of 18 months, he was diagnosed with cryptogenic focal epilepsy with complex focal seizures, severe developmental retardation, and optic atrophy. At this age, he presented with almost no psychomotor development. Most medications failed to control seizures or had severe side effects. Currently, seizures are under control with topiramate, pregabalin and lamotrigine.

On MRI at the age of 2 ½ years, there was thinning of the *corpus callosum* as well as reduction of gray and white matter with increased ventricle and subarachnoid liquor spaces. A follow-up MRI at the age of 5 ½ years revealed cerebral atrophy, especially in the frontal and temporal regions (for detailed clinical history see Supplementary Complete Clinical Information, as well as Supplementary Table S1, Supplementary Figs. S2 and S3).

Exome sequencing was performed in 2014 from a DNA sample isolated from peripheral blood of the affected individual. Libraries were constructed using Agilent All Exon V5 enrichment and next-generation sequencing (100 bp paired-end) was performed using the lllumina HiSeq technology. Within the targeted coding exons, the average depth of coverage was 60-fold with 90% of target sequence covered at least 10-fold. Variant calling in genes known to be causatively related to epileptic encephalopathies revealed a novel missense variant located in exon 10 of the *GABRA1* (gamma-aminobutyric acid type A receptor alpha1 subunit) gene: c.995C > T p.(Ala332Val). The variant introduces an amino acid exchange of a highly conserved alanine residue to valine in TM3 (A332V) and is not listed as rare minor allele in reference datasets like gnomAD^24^. *In silico* prediction using different algorithms, such as MutationTaster and CADD, indicated a high probability of pathogenicity^25–27^. While conventional DNA sequencing confirmed the presence of the variant in the affected individual, it was shown to be absent in DNA isolated from both parents, leading to a genetic diagnosis compatible with early infantile epileptic encephalopathy, caused by a *de novo* missense variant in *GABRA1*.

### Structural analysis and localisation of the variant

**Figure 1:**
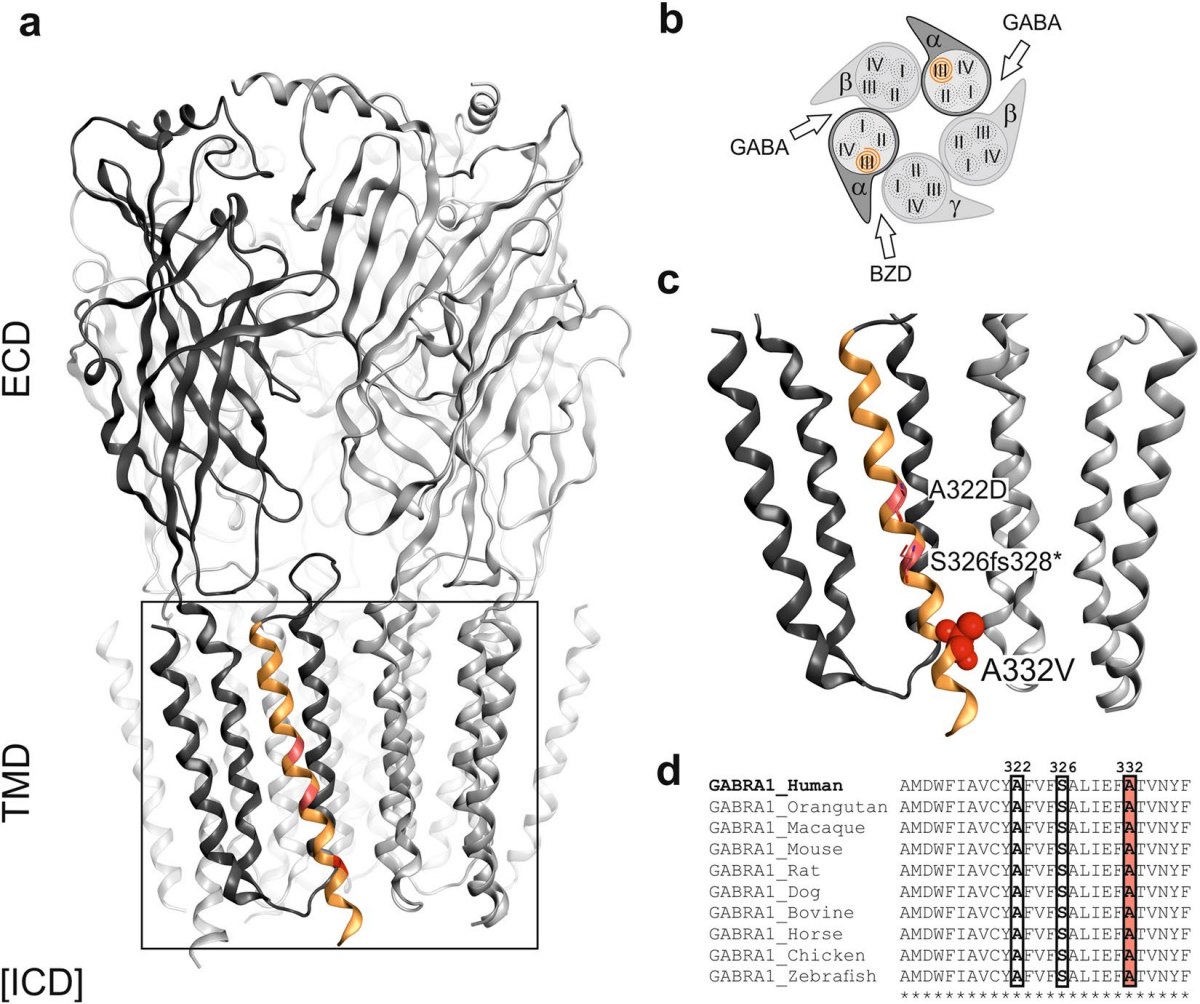
GABA_A_ receptor structure highlighting the A332V *de novo* variant. Panel (**a**) depicts an α1β3γ2 GABA_A_ receptor (the cryo-EM structure with PDB ID 6HUP^47^). The schematic representation of the pentamer in panel (**b**) depicts a canonical α1β3γ2 receptor consisting of two α, two β and one γ subunit, where the TMD helices are numbered 1–4 (TM3 is highlighted). The binding sites for GABA and benzodiazepines (BZD) are indicated by arrows. Panel (**c**) shows a detailed view of the TMD region (M3 helix) where A332V, as well as A322D and S326fs328* are located. A332 is shown in a red atom view representation. In panel (**d**), a partial sequence alignment highlighting the 3 variants in TM3 of GABA_A_ receptor α1 subunit in humans with α1 subunit in other species, shows the levels of conservation (highlighted in red the high conservation of the A332 among the different species).

Disease associated variants are found in all three domains of the α1 protein, without any tendency to cluster in any particular region^22^. The α1 variant A332V described here is located in TM3 near the subunit interface. Two other missense variants associated with epilepsy have been also identified within this helix (A322D and the frame-shift S326fs328*). The TM3 is highly conserved between different species (see Fig. 1d) and especially the position 332 is also highly conserved between different subunits (Supplementary Fig. S4). This helix is part of a subunit-subunit interface. Even a rather conservative exchange in this highly conserved position is expected to impact on protein function.

### *In vitro* expression, fluorescence western blot and immunostaining

Several studies reported that mutations in GABA_A_ receptor subunits can impair cellular processes such as protein trafficking and expression^18^. We examined the protein levels and cellular localization of the mutated GABA_A_ receptor in transfected HEK293 cells. As α1β3γ2 receptors are highly expressed in the brain, we studied this subtype in the HEK293 cells.

We used western blot analysis in order to estimate the protein expression levels of mutated and wild-type receptors. Due to the membrane preparation method it was not possible to differentiate in the western blot between surface and intracellular receptors.

**Figure 2:**
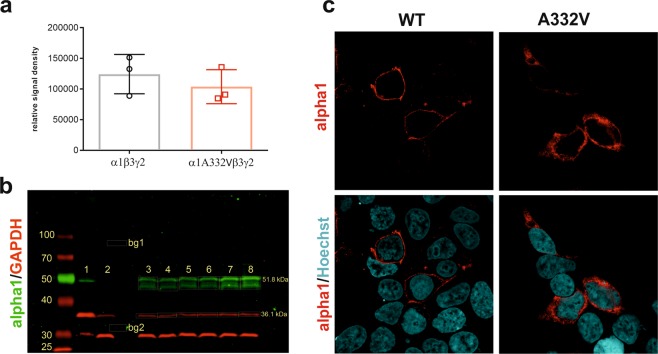
Protein expression of α1 in HEK293 cells. (**a**) Expression of total α1 protein was detected in HEK293 cells transfected with wild-type or mutant human α1β3γ2 cDNAs (ratio 1:1:5). Protein levels were normalised to GAPDH expression. (**b**) Representative western blot analysis (n = 3). 1: 20 µg mouse Hippocampus, 2: 20 µl untransfected HEK293 cells, 3–5: 20 µl HEK293 α1A332Vβ3γ2, 6–8: 20 µl HEK293 α1β3γ2; bg1: background signal for green channel (α1), bg2: background signal for red channel (GAPDH). (**c**) Immunostaining of surface α1 expression (red). Both mutated and wild-type subunits appear at cell surfaces when co-expressed with β3 and γ2 subunits.

As shown in Fig. 2a, we did not observe a significant difference between total wild-type and mutant α1 protein expression level (p = 0.45). This indicates that the mutated subunit is expressed at a similar level as the wild-type subunit.

Because in western blot no difference in total protein was detected we performed an immunostaining on transfected HEK293 cells to see if the cellular distribution of mutated and wild-type protein is similar. The results showed that wild-type and mutated α1 subunits can be detected at the cell surface to a similar amount and no significant intracellular staining was observed (see Fig. 2c).

### Radioligand displacement assays

Benzodiazepines bind in the ECD between α1 and γ2 subunits^28^. Therefore, radioligand displacement assays with diazepam can be used to assess receptor assembly. We used α1β3γ2 transfected HEK293 cells with wild-type or mutated α1 and displaced 3H-flunitrazepam with varying concentrations of diazepam (Supplementary Fig. S5).

**Figure 3:**
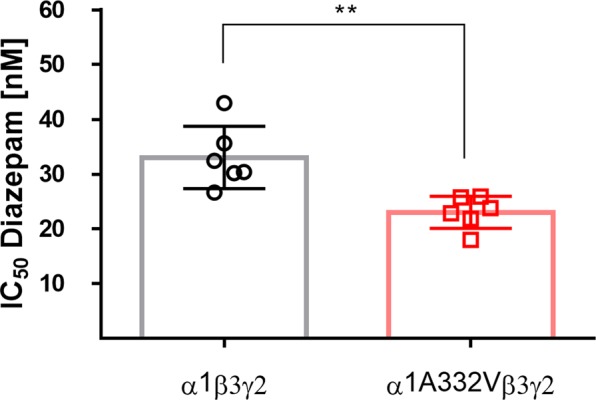
^3^H-flunitrazepam displacement in transfected HEK293 cells. ^3^H-flunitrazepam displacement with increasing concentrations of diazepam was performed in HEK293 cells transfected with human α1β3γ2 cDNA with either wild-type α1 or mutant α1A332V. Values depict mean ± SEM from six individual experiments. IC_50_ values for α1β3γ2 and for α1A332Vβ3γ2 are 33 nM and 23 nM, respectively. Statistically significant differences were determined by two-tailed students *t*-test, where p < 0.05; **p < 0.01.

Radioligand binding was observed in both wild-type and mutated receptors, thus it appears that the mutated subunit is inserted in the receptor next to the γ2 subunit as is the case for the α1 subunit in wild-type receptors. The calculated IC_50_ values differed significantly (p = 0.003) between wild-type and mutated receptors (33 and 23 nM, respectively, Fig. 3). The variant seems to increase the affinity of diazepam to the receptor.

### Functional (two-electrode voltage clamp electrophysiology) data

**Figure 4:**
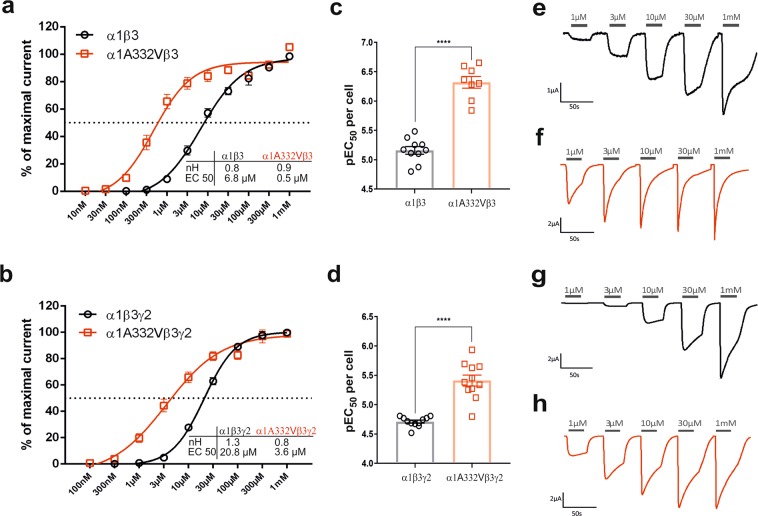
GABA dose response relationships in wild-type and mutated receptors. (**a**,**b**) GABA dose response curves of all four wild-type and mutated receptors. Data were fitted to a sigmoidal dose response with a variable slope and points are depicted as mean ± SEM. Each data point represents experiments from n = 7–12 oocytes from ≥2 batches. (**c**,**d**) The column graphs depict the pEC_50_ values of α1A332Vβ3, α1β3, α1A332Vβ3γ2 and α1β3γ2 receptors obtained by fitting data of each cell individually. Statistically significant differences were determined by two-tailed students *t*-test, where p < 0.05; ****p < 0.0001. (**e**,**f**) Representative traces from electrophysiological recordings in α1β3 (**e**) and α1A332Vβ3 (**f**) r**e**ceptors. (**g**,**h**) Representative traces from electrophysiological recordings in α1β3γ2 (**g**) and α1A332Vβ3γ2 (**h**) receptors.

Upon agonist binding to the ECD and activation of the GABA_A_ receptor, conformational changes occur that lead to channel opening. In order to assess whether the variant can have an impact on agonist effects, we expressed recombinant receptors containing human α1A332Vβ3 or α1β3 in *Xenopus laevis* oocytes either alone or in combination with γ2, corresponding to an extrasynaptic and a synaptic subunit combination. After protein expression, we recorded whole cell GABA-induced currents with two-electrode voltage clamp electrophysiology. GABA dose response curves revealed a significant left shift (Fig. 4a,b). Interestingly, GABA activates α1A332Vβ3 receptors with higher potency than wild-type receptors inducing effects already at nanomolar range (13% of maximum current at 100 nM) with EC_50_ values of 6.8 μM for wild-type receptors and 0.5 μM for mutated receptors (Fig. 4a,c). We also compared GABA dose response curves of α1β3γ2 with α1A332Vβ3γ2 receptors and also observed a significant left shift of the curve with EC_50_ values of 20.8 μM and 3.6 μM, respectively (Fig. 4b,d). To ensure the incorporation of the γ2 subunit, we tested if γ2- containing receptors are sufficiently modulated by diazepam (~200% at 1 µM diazepam). The relative populations of α1β3 receptors and α1β3γ2 receptors cannot be quantified readily, but both the diazepam modulation and the different trace characteristics (Fig. 4e–h) demonstrate that the cells express a sufficient amount of the ternary receptor. Moreover, absolute currents elicited by 1 mM GABA are significantly higher in α1β3γ2 compared to α1A332Vβ3γ2 receptors, whereas no statistically significant difference was observed between α1β3 and α1A332Vβ3 (Supplementary Fig. S6).

We next determined whether the variant changed the maximum efficacy of GABA by estimating the maximum open probability of GABA similar to previous studies^29^. As shown in previous studies, the endogenous agonist GABA cannot activate the receptors to their maximal obtainable open-state probability (P_O(max)_)^30^. Measuring the maximal efficacy of GABA in presence of an allosteric modulator is an established pharmacological method of estimating P_O(max)_, and is utilised in the absence of single-channel recordings^30–32^. This technique enables us to determine whether the variant changes these intrinsic activation properties of the receptor.

**Figure 5:**
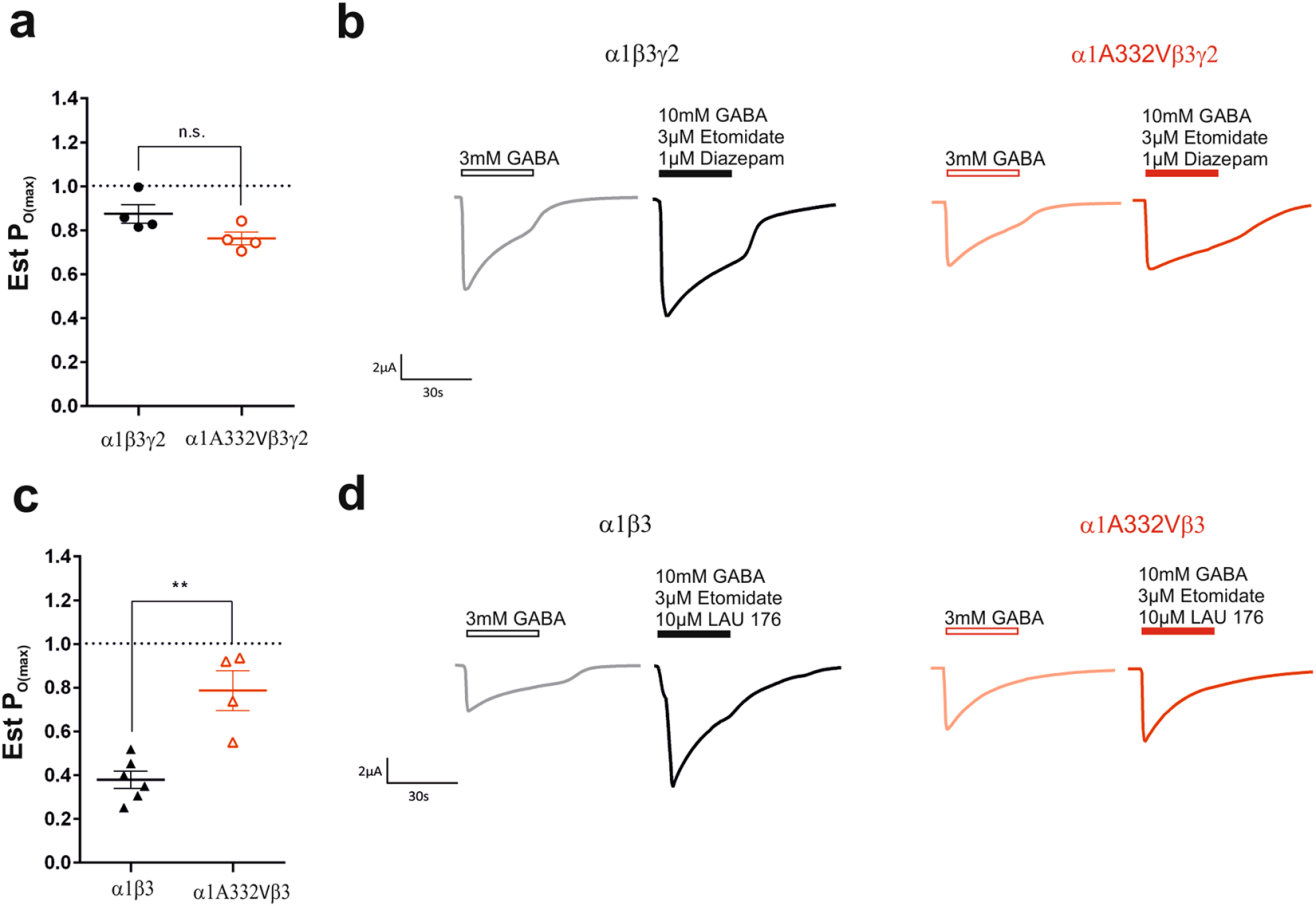
(**a**) Est. P_O(max)_ values of α1β3γ2 and α1A332Vβ3γ2 receptors. The values were determined by dividing the current elicited by 3 mM GABA by the current elicited by 10 mM GABA, 1 µM diazepam, and 3 µM etomidate and corrected for the reference 3 mM GABA current. Lines and error bars represent the mean ± SEM of 4 individual cells. (**b**) Representative traces from electrophysiological recordings in α1β3γ2 and α1A332Vβ3γ2 receptors after application of reference 3 mM GABA and 10 mM GABA, 1 µM diazepam, and 3 µM etomidate, respectively. (**c**) Est. P_O(max)_ values of α1β3 and α1A332Vβ3 receptors. The values were determined by dividing the current elicited by 3 mM GABA by the current elicited by 10 mM GABA, 10 µM LAU 176, and 3 µM etomidate and corrected for the reference 3 mM GABA current. Lines and error bars represent the mean ± SEM of 4–6 individual cells. (**d**) Representative traces from electrophysiological recordings in α1β3 and α1A332Vβ3 receptors after application of reference 3 mM GABA and 10 mM GABA, 10 µM LAU 176, and 3 µM etomidate, respectively. Statistically significant differences were determined by two-tailed students *t*-test, where p < 0.05; **p < 0.01.

We thus compared the Est. P_O(max)_ at α1A332Vβ3γ2 and α1β3γ2 receptors (Fig. 5a,b). We expected, as observed previously, that the co-application of GABA with etomidate and diazepam would open the receptors with a high probability^29^. Therefore, we applied 3 mM GABA as a reference at α1A332Vβ3γ2 and α1β3γ2 receptors and then co-applied 10 mM GABA with 3 μM etomidate and 1 μM diazepam (Fig. 5a,b). This was performed in order to shift as many receptors as possible to the open state. A high Est. P_O(max)_ close to 1 was observed at both wild-type and mutated receptors with no significant difference. Next, we similarly applied 3 mM GABA as a reference at α1A332Vβ3 and α1β3 receptors and then co-applied 10 mM GABA with 3 μM etomidate and 10 μM LAU 176 (Fig. 5c,d). LAU 176 is an established, highly efficacious, positive allosteric modulator of α1β3 receptors, acting primarily via the extracellular domain^33,34^. We observed a significant difference between wild-type and mutated receptors, with the later resulting in a higher Est. P_O(max)_ (Fig. 5c,d).

**Figure 6:**
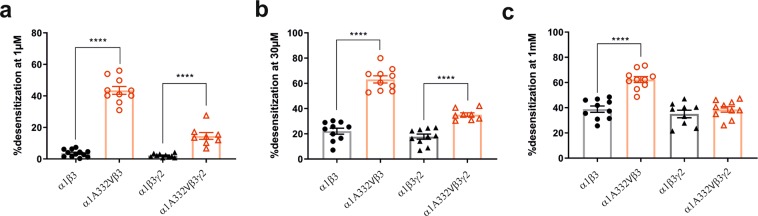
Extent of desensitization of wild-type and mutated receptors. The extent of desensitization was determined at 1 µM (**a**), 30 µM (**b**) and 1 mM GABA (**c**) as described in the methods (peak to 15 s) in all four receptor types. Statistically significant differences were determined by two-tailed students *t*-test, where p < 0.05; ****p < 0.0001. Bars are presented as mean ± SEM. A comparison based on comparable level of activation, rather than on GABA concentration, is provided in Supplementary Fig. S7, while representative traces are depicted in Supplementary Fig. S8.

To obtain more insight into the impact of α1A332V on the receptor properties, we analysed the extent of desensitization in wild-type and mutated receptors at 1 µM, 30 µM and 1 mM GABA. α1A332Vβ3 receptors exhibited a significantly more pronounced desensitization with 40% at 1 µM GABA and about 60% at 30 µM and 1 mM GABA compared to wild-type receptors (Fig. 6). Similarly, the extent of desensitization differed significantly in α1A332Vβ3γ2 at 1 µM and 30 µM GABA, however, at 1 mM GABA there is no difference in the extent of desensitization between α1A332Vβ3γ2 and α1β3γ2 (Fig. 6c).

The receptors with the mutated α1 subunit are activated to a greater extent at the same GABA concentration due to the left shift (Fig. 4), and thus, the extent of desensitization might just reflect this left shift^35^. To clarify, we also compared extent of desensitization at comparable level of activation or % of Imax (Supplementary Figs. S7 and S8). The extent to which the mutated receptors desensitize is higher also at comparable activation level.

**Figure 7:**
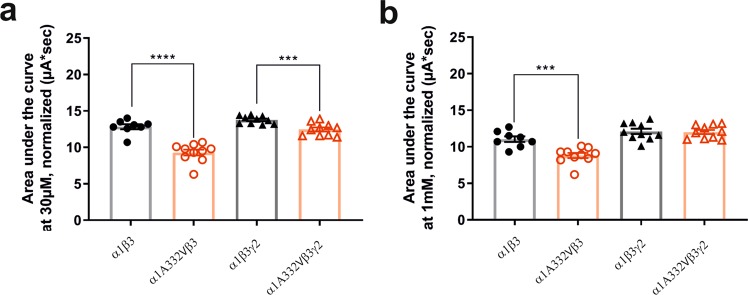
Area under the curve of wild-type and mutated receptors. Area under the curve was calculated at 30 µM (**a**) and 1 mM GABA (**b**) in all four receptor types. Statistically significant differences were determined by two-tailed students *t*-test, where p < 0.05; ***p < 0.001; ****p < 0.0001. Bars are presented as mean ± SEM.

Additionally, as an approximate measure for charge transfer in a GABA elicited event, the area under the curve (AUC) was determined at 30 µM and 1 mM GABA in all four receptor types (Fig. 7). The results revealed that at both concentrations the AUC of the binary mutated receptor is significantly smaller than the binary wild-type receptor. A significant difference at 30 µM can be also observed in α1β3γ2 and α1A332Vβ3γ2 receptors, whereas at 1 mM no difference is seen (Fig. 7).

## Discussion

The identification of a - hitherto unreported - variant of unknown significance in a known disease associated gene is a frequently encountered result during clinical exome sequencing in infants with a suspected hereditary condition. Like in the case described in this paper, segregation analysis in healthy parents can determine whether the variant arose *de novo* and therefore represents formally a good candidate for being the disease-causing variant. Algorithms for predicting the probability whether a missense variant introduces a pathogenic amino acid exchange, such as MutationTaster or CADD, have evolved into powerful tools to streamline the process of assessing the significance of a novel variant^25–27^. However, without functional studies, there will always be at least some degree of uncertainty in the final assessment. In the case reported here, the severe early-onset clinical manifestation of the affected individual prompted us to investigate the functional consequence of the identified variant (A332V).

A332V is localised in helix 3 of the transmembrane domain (TMD) of the α1 subunit. The TMD is highly conserved among different GABA_A_ receptor subunits and even within the Cys-loop receptor family: nAChR, 5-HT_3A_R, glycine receptors and even bacterial homologues show extremely high structural conservation on a basis of as little as about 15% sequence identity^17^. Consistent with this, many variants in the TMD cause a molecular phenotype that can range from altered channel properties with regular expression to reduced functional expression due to misfolding or protein retention in the ER^19,20^. The case of α1A322D has been studied in considerable detail. In this case, ER retention (likely due to misfolding) results in very low receptor expression, and thus, a clear “loss of function” phenotype.

Here we performed an *in vitro* characterisation of the A332V variant. Expression and forward trafficking to the cell surface appears unchanged in HEK293 cells as evidenced by western blot results indicating that the amount of total α1 protein is similar to wild-type, and further supported by immunocytochemical stainings indicating regular trafficking to the cell surface.

The radioligand displacement assays in HEK293 cells indicate that the α − γ interface in recombinant α1A332Vβ3γ2 receptors is regularly built because the binding of benzodiazepines to the α1(+) γ2(−) interface is not impaired. However, the IC_50_ values of diazepam-displacement differ by one order of magnitude, with diazepam binding with higher affinity to the mutated receptor. Nevertheless, this shift might not be pharmacologically relevant *in vivo*.

Functional analysis was performed in *Xenopus laevis* oocytes, using cells injected with α1A332V and β3 mRNA as well as cells with the ternary α1A332V, β3 and γ2 combination. The former cells express an extrasynaptic receptor subtype, while the ternary mix of mRNA yields mixed pools αβ and αβγ receptors^36,37^. Robust GABA elicited currents indicate also in this recombinant system regular expression and forward trafficking.

The mutated α1 subunit confers changes in GABA potency, where the mutated subunit induces an apparent left shift of the GABA dose response compared to wild-type for both tested subunit combinations. We sought to also investigate channel opening and desensitization characteristics. Since the maximum current amplitude may be underestimated by application of GABA alone, we estimated the maximum open probability of wild-type and mutated receptors as described previously^29,31,32^. Similar to Absalom *et al*. we observed a high Est.P_O(max)_ in wild-type γ-containing receptors. For the mutated receptors no significant difference to WT was observed. For the case of α1β3 receptors GABA does not seem to be a full agonist, since the application of established allosteric modulators resulted in more pronounced enhancement of the current elicited by a saturating GABA concentration. In contrast, this increase in efficacy is not observed in α1A332Vβ3 receptors resulting in a significantly higher Est.P_O(max)_ compared to wild-type receptors in response to a saturating GABA concentration. α1β3 receptors are considered to contribute to an extrasynaptic pool of receptors which mediate tonic inhibition^37^. Typically, extrasynaptic receptors are activated by low concentrations of ambient GABA, causing long lasting currents which tend to be smaller than currents produced by synaptic receptors. A characteristic of this pool of receptors is that due to their different intrinsic properties compared to synaptic receptors they can confer seemingly magnified modulatory efficacy, an outcome often observed with several allosteric modulators^31^. It is interesting to note that the mutation impacts on GABA efficacy for α1A332Vβ3, but not for α1A332Vβ3γ2.

Receptor desensitization is also altered. Due to the faster desensitization, the total charge transfer per GABA elicited event is reduced as evidenced by the area under the curve of the whole cell current traces. Comparing desensitization levels at comparable levels of activation indicates that the mutant accelerates desensitization in both the tested subunit combinations. This could be rationalised from the proximity of the mutation to the desensitization gate in the lower TMD^38^.

In summary, subunit folding, receptor assembly and forward trafficking appears to be unchanged in HEK cells and *Xenopus* oocytes. In contrast, channel properties are changed considerably, and in ways that depend on subunit composition. The α1 subunit contributes to multiple receptor subtypes expressed by neurons, including phasically active α1βγ2 and tonically active α1β, α1βδ or α1α6βδ receptors, just to name a few. The altered GABA efficacy, sensitivity and desensitization characteristics will impact on both phasic and tonic currents in neurons in ways that cannot be predicted based on the data presented here, because the actual impact of increased sensitivity at low GABA concentrations and accelerated desensitization already at moderate concentrations will depend on the precise neuronal events: At extrasynaptic receptors the influence of desensitization may lower the effective inhibitory tone in spite of the higher GABA potency, and thus contribute to a shift in seizure threshold. On the other hand, the left shifted GABA response may lead to altered phasic inhibition, where inhibitory postsynaptic events may occur excessively or due to ambient GABA.

A332V should be seen as a “molecular change of function” variant which induces an increased sensitivity to low GABA concentrations in combination with accelerated desensitization and some impact on agonist efficacy that is more pronounced in α1β3 receptors. What can be extrapolated to the possible role of the missense variant in the heterozygous carrier? Here we tested receptors in which all subunits are either wild-type, or mutated. It has been shown recently that functional alterations can be remarkably different if a pentamer contains one wild-type and one mutated subunit, and that even the position of the mutated subunit severely impacts on receptor function^29^. Given that the affected individual is heterozygous, conclusions from the molecular findings here to the onset and development of symptoms are impossible. The net effects of the heterozygous variant can be investigated in more detail only in transgenic animals, or patient derived organoid or spheroid cell culture models. To discern under which circumstances the mutant will contribute to reduced, or enhanced GABA responses requires a broad variety of experiments with *in vitro* and *in vivo* preparations that are able to recapitulate the presence of one wild type and one mutated gene, the wide diversity of α1 containing receptor species, and their temporal expression patterns in the time after birth when the *GABRA1* expression gets gradually upregulated.

In spite of all these limitations, it still is possible to speculate on possible mechanisms by which the observed molecular phenotype could induce some aspects of the syndrome and specifically could be driving the seizure phenotype. The onset of symptoms can be seen as highly consistent with the postnatal up-regulation in *GABRA1* expression in the brain. Seizures are indicative of an excitation/inhibition imbalance. A direct effect by a dramatic loss of function at the receptor level cannot be predicted based on the *in vitro* data, while some mixture of loss and gain of function would be plausible. A secondary loss of receptors due to enhanced internalization that develops over time would be a plausible candidate in case the gain of function occurs as a dominant consequence of the altered mix of molecular properties. In fact, if GABA_A_ receptors are chronically stimulated with agonists or positive modulators, increased receptor turnover and subsequent changes in gene expression have been consistently observed, suggesting that excessive channel activity leads to compensatory changes^39^. The left shifted GABA response we observe here could thus drive a secondary downregulation of the affected and other subunits. This hypothesis is probably the most plausible, but impossible to validate with *in vitro* experiments.

Overall, we can conclude that the A332V missense variant in the α1 subunit impacts receptor function at the level of GABA and benzodiazepine function, and on desensitization characteristics, which might drive a broad range of adaptive cellular responses.

## Methods

All methods were carried out in accordance with relevant guidelines and regulations of the Medical University of Vienna, Austria.

### Clinical information

Clinical information and family history were collected using records submitted by the referring clinicians. Informed consent was obtained from the patient’s parents in accordance with the regulations of local ethics review boards of the Medical University of Vienna, Austria.

### Next-generation sequencing

Exome sequencing was performed in 2014 from a DNA sample isolated from peripheral blood of the affected individual. Libraries were constructed using the Agilent SureSelect All Exon V5 kit (Agilent, Santa Clara, CA, USA) and sequenced using the lllumina HiSeq sequencing system (Illumina, San Diego, CA, USA) with 100 bp paired-end reads. All library preparation and sequencing steps were performed at GATC Biotech AG (now: Eurofins Genomics; Constance, Germany). Sequences were analysed at the Neuromuscular Research Department using an in-house developed bioinformatics pipeline starting from fastq files. In brief, reads were mapped to the human genome reference sequence (build hg19) using the BWA-MEM algorithm^40^. Within the targeted coding exons, the average depth of coverage was 60-fold with 90% of target sequence covered at least 10-fold. Variant calling was performed using the GATK Haplotype Caller^41^. Variant filtration was performed with ANNOVAR and exported to Excel spreadsheets^42^. The final data analysis was done using minor allele frequencies from reference datasets (1k genomes, ESP, ExAc, gnomAD) for genes known to be causatively related to epileptic encephalopathies, which revealed a so far unreported variant in the *GABRA1* (gamma-aminobutyric acid type A receptor alpha1 subunit) gene: hg19 chr5:g.161322810C > T; NM_000806.5:c.995C > T p.(Ala332Val). Different algorithms were used to predict potential pathogenicity of the identified variant, including MutationTaster (P > 0.999) and CADD (Phred = 34)^25–27^.

Segregation was analysed by PCR (forward primer: 5′- TTTCCTTTTGTTCAAGTAGGCTGT-3′; reverse primer: 5′-TTCTGCATACTCCATCCAATACC-3′, for amplifying a 390 bp-fragment comprising of exon 10 and flanking intron boundaries), followed by conventional Sanger DNA sequencing in the affected individual and both parents.

### Cloning

Cloning of the mutated human α1 cDNA was performed using a human α1-pCI construct and the Q5 Site-Directed Mutagenesis Kit (New England Biolabs) following manufacturer’s instructions with the following primers: 5′-ATTGAGTTTGtCACAGTAAC-3′ and 5′-CAGAGCTGAGAACACAAAG-3′. The small letter indicates the base changed by mutagenesis leading to an exchange of alanine at position 332 to valine in the protein.

### HEK293 cell transfection and radioligand binding

Transfection of HEK293 cells with a combination of human wild-type or mutant α1β3γ2 cDNAs (ratio 1:1:5) and displacement of radioactively labelled ^3^H-flunitrazepam with different concentrations of diazepam (from 1 nM, 3 nM, 10 nM, 33 nM, 100 nM, 333 nM, 1 µM to 3 µM) was performed as described previously^43^.

### Membrane preparation and protein determination

Membranes of HEK293 cells transfected with either wild-type or mutant human α1β3γ2 cDNAs (ratio: 1:1:5) were prepared as described previously^44^. Protein concentrations were determined using the Pierce™ BCA protein assay kit (ThermoFisher Scientific) following manufacturer’s protocol.

### SDS-PAGE, Western blot and fluorescence detection

Samples for western blot were prepared as described previously^44^. 20 µg of samples and 0.5 µl of protein marker were separated on a gel according to standard Laemmli method. Gels were blotted semi-dry on pre-wetted polyvinylidene fluoride membranes. Membranes were blocked in blocking buffer (5% milk/PBS without Tween 20) o/n at 4 °C. They were then incubated with 0.5 µg/ml primary antibody in blocking buffer (5% milk/PBST) for 24 h at 4 °C^45^. Afterwards, membranes were incubated with 0.5 µg/ml primary anti-GAPDH mouse antibody in blocking buffer for 24 h at 4 °C and washed 4 × 5 min with washing buffer (1.5% milk/PBST). Membranes were incubated with secondary antibodies diluted 1:10 000 in washing buffer (Alexa Fluor® 680-conjugated goat anti-mouse light chain, Jackson ImmunoResearch and IRDye® 800CW-conjugated donkey anti-rabbit, LI-COR® Biosciences) for 1 h at room temperature. After incubation, membranes were washed again 4 × 5 min with washing buffer. Membranes were scanned at 700 and 800 nm using the Odyssey System (LI-COR® Biosciences). Band intensities were analysed with the Image Studio software version 5.2 for Windows (LI-COR® Biosciences). A complete western blot is shown in Fig. 2b.

### Immunocytochemistry

HEK293 cells transfected with a combination of human wild-type or mutant α1β3γ2 cDNAs (ratio 1:1:5) were fixed with fresh 4% PFA followed by 2x washing with 1x PBS. Cells were then incubated for 1 h at RT in blocking solution (10% normal donkey serum (NDS), 5% BSA, 0.3% Triton X-100/PBS). After blocking, the cells were treated with 0.65 µg/ml primary antibody in buffer (5% NDS, 2% BSA, 0.3% Triton X-100/PBS) for 16 h at 4 °C^45^. After washing the cells 3 × 20 min in PBS, immunoreactivities were revealed using anti-rabbit (1:300) secondary antibodies (Jackson ImmunoResearch) conjugated with Cy3 fluorophores diluted in 2% BSA in PBS. Nuclei were stained with Hoechst. After the final washes (3 × 40 min) the coverslips with the stained cells were mounted on microscopy glass slides with glycerol gelatine. Imaging was executed on a Zeiss 780LSM laser-scanning microscope and assembled in CorelDraw X7 (Corel Corporation).

### Two-electrode voltage electrophysiology

All steps were performed as reported previously^33^. In brief, cloned pCI vectors encoding for human GABA_A_ receptor subunits α1, α1A332V, β3 and γ2 were linearised, transcribed and purified in order to generate mRNA. For the microinjection, the RNA of the α1/α1A332V -β3 receptor combination was mixed at 1:1 ratio and for the α1/α1A332V-β3γ2 receptor at 1:1:5 ratio with a final concentration of 56 ng/µl. Mature *Xenopus laevis* oocytes were obtained in full accordance with all rules of the Austrian animal protection law and the Austrian animal experiment by-laws which implement European Directive 2010/63/EU into Austrian law. Stage 5–6 oocytes were then digested and defolliculated as described previously^33^. The cells were injected with an aqueous solution of mRNA (2.8 ng/oocyte) and incubated for 2–3 days at 18 °C before electrophysiological recordings. For current measurements the oocytes were impaled with two microelectrodes (1–3 MΩ) filled with 2 M KCl. The oocytes were constantly washed by a flow of 6 ml/min NDE (96 mM NaCl; 2 mM KCl; 1 mM MgCl_2_ × 6H_2_O; 5 mM Hepes; 1.8 mM CaCl_2_ × 2H_2_O; pH 7.5) that could be switched to NDE containing GABA. GABA was applied for 20 seconds and between two applications oocytes were washed in NDE for up to 15 min to ensure full recovery from desensitization.

For estimating the maximal open probability Est. P_O(max)_, experiments were conducted similar to Absalom *et al*.^29^. 3 mM GABA was used as a reference concentration and was applied two times at the beginning of the experiment. For αβ containing receptors, a solution consisting of 10 mM GABA, 3 µM etomidate and 10 µM LAU 176 was applied for 30 seconds^33,34^. For αβγ containing receptors 1 µM diazepam was used instead of LAU 176. As shown in Absalom *et al*., cells were washed 10–15 min between the applications. Peak currents were normalised to the mean of the two 3 mM GABA applications. All recordings were performed at room temperature at a holding potential of −60 mV using a Dagan TEV-200A two-electrode voltage clamp (Dagan Corporation). Data were digitised, recorded and measured using an Axon Digidata 1550 low-noise data acquisition system (Axon Instruments). Data acquisition was performed using pCLAMP v.10.5 (Molecular Devices™).

### Data analysis

For the radioligand binding assays, IC_50_ values were derived from the displacement curve calculated as nonlinear fit with a variable hill slope using the following formula and bottom = 0%, top = 100%:

$${\rm{Y}}={\rm{Bottom}}+\frac{{\rm{Top}}-{\rm{Bottom}}}{1+{10}^{(({\rm{LogIC}}50-{\rm{X}})\ast {\rm{Hillslope}})}}$$

Data obtained from electrophysiological experiments were analysed using GraphPad Prism v.7.00 and plotted as concentration-response curves. These curves were normalised and fitted by non-linear regression analysis to the equation, where logEC_50_ is the X value when the response is half way between top and bottom y values, and nH is the Hill coefficient.

$${\rm{Y}}={\rm{Bottom}}+\frac{{\rm{Top}}-{\rm{Bottom}}}{1+{10}^{(({\rm{LogEC}}50-{\rm{X}})\ast {\rm{Hillslope}})}}$$

Data are given as mean ± SEM from at least five oocytes of two and more oocyte batches.

The desensitization of the receptor and the respective area under the curve (AUC) for each trace were calculated for the region between the maximal current amplitude and the residual current amplitude reached 15 sec after maximum. Desensitization was measured at currents induced by 1 µM, 30 µM and 1 mM GABA for all receptors. The extent of desensitization was determined as given:

$${\rm{Desensitization}}[ \% ]=(1-\frac{{\rm{I}}residual}{{\rm{I}}peak})\times 100$$

*I*_*peak*_ is the agonist-induced peak current and *I*_*residual*_ the residual of this current 15 seconds after the peak amplitude^46^.

For calculating the area under the curve, currents induced by 30 µM and 1 mM GABA in all four receptor types were taken into account. The traces were first normalised so that all points of the current response span the range 0 to 1. The normalisation function is as follows, where y_min_ is the smallest and y_max_ is the largest value in the trace:

$$f\,(y)=-(1-\frac{(y-y\,{\min })}{(y\,{\max }\,-y\,{\min })})$$

The areas under the curve confined by the baseline, the normalised maximum and the normalised residual current 15 sec after maximum were determined by the “Area” function of Clampfit 10.7.03.

Statistical significance for all analyses was calculated using a one sample *t*-test. P-values of <0.05 were accepted as statistically significant.

## Supplementary information

Supplementary Information.

## Data Availability

The datasets generated and/or analysed during the current study are available from the corresponding author upon request.
