# Supplementary material for: A novel de novo variant of GABRA1 causes increased sensitivity for GABA in vitro
Source: Sci Rep. 2020 Feb 11;10:2379. doi: 10.1038/s41598-020-59323-6 (PMC7012862; doi:10.1038/s41598-020-59323-6)

Supplementary Information

A novel *de novo* variant of *GABRA1* causes increased sensitivity for GABA *in vitro*

Friederike Steudle (1)§, Sabah Rehman (2)§, Konstantina Bampali (2)*, Xenia Simeone (2), Zsofia Rona (3), Erwin Hauser (3), Wolfgang M. Schmidt (4), Petra Scholze (1), Margot Ernst (2)

(1) Department of Pathobiology of the Nervous System, Center for Brain Research, Medical University Vienna, Vienna, Austria
(2) Department of Molecular Neurosciences, Center for Brain Research, Medical University Vienna, Vienna, Austria
(3) Landesklinikum Thermenregion Mödling, Department of Pediatrics, Mödling, Austria
(4) Neuromuscular Research Department, Center for Anatomy and Cell Biology, Medical University of Vienna, Vienna, Austria

§ These authors have contributed equally to this work.

*Corresponding author: konstantina.bampali@meduniwien.ac.at, phone number: +43 1 40160 34252

Supplementary Complete Clinical Information

The affected individual was born in Vienna at 39+3 weeks of gestation (normal spontaneous delivery) from an uneventful (di-zygotic) twin-pregnancy as the third child of healthy parents of Chechen descent. His family history was unremarkable, all siblings including his twin sister are healthy.

The first epileptic seizure (*status epilepticus*) was observed at an age of 2 ½ months and included tonic flection of the arms, extension of the legs with head deviation and rhythmic head movements, as well as subsequent apnea in waking state. An EEG was initially normal with no focal or generalized epileptic discharges, but a generally slow background activity with dominant delta-theta waves.
Initial antiepileptic treatment was started with valproate, with no effect, and then supplemented with levetiracetam, which was also unsuccessful. Also most other following medications failed to control seizures after 3 months, such as zonisamide, or had severe side effects such as leucopenia on vigabatrin therapy or hyponatremia on treatment with oxcarbazepine. Currently, seizures are under control with topiramate, pregabalin and lamotrigine (antiepileptic treatment history is summarized in Table S1).
At age of 18 months, he was diagnosed with cryptogenic focal epilepsy with complex focal seizures, developmental retardation, movement disorder (suspected cerebral palsy), and optic atrophy. At this age, he presented with almost no psychomotor development.
At the age of 8 years, he was diagnosed with severe mental retardation, which could not be fully explained by seizures alone (only few epileptic potentials in EEG).

Imaging

On the first brain MRI at the age of 2 ½ years, there was cerebral brain atrophy, thinning of the *corpus callosum* as well as reduction of gray and white matter with increased ventricle and subarachnoid liquor spaces. A repeated MRI at the age of 5 ½ revealed a similar picture of a cerebral brain atrophy, especially in the frontal and temporal regions. The cerebellum was on both occasions completely normal, no focal lesions were detected (Figure S2).

Epilepsy and EEG

At the age of 2 years, he developed in EEG sharp waves and spike wave complexes mainly temporal and occipital on the left side with a multifocal focus. The background pattern was generally slower with mainly theta and delta waves. In the sleep EEG sleep stages were there and the temporal and the focally appearing temporal and occipital sharp waves have no clinical seizure correlate (Figure S3).

In a sleep EEG conducted at the age of 2, all sleep stages were present. Multifocal sharp waves and spike wave discharges were frequent in sleep as well as in wake state especially over the temporo-occipital region on the left side. Electrophysiological seizures had no clinical epileptic correlate with

two years of age. They were often followed with repetitive slow delta waves discharges. He never showed photosensitivity.

Clinical features

The affected individual is currently 13 years old. He has severe muscle hypotonia. His facial features are dysmorphic with brachycephaly and microcephaly. He has a percutaneous endoscopic gastrostomy (PEG) button since the age of 5 years old because of recurrent aspirations and insufficient swallowing. His appendix was removed and he had a cholecystectomy because of biliary sludge and small stones. Both hips were dislocated and surgery took place at the age of 11 years to solve the subluxation.

Neuropsychological Profile

At the age of 12, the affected individual has a severe psychomotor retardation with a developmental level of a 5 months old infant. He can laugh and he reacts when parents or caregivers call his name, but he cannot follow or grab objects, but can fixate shortly, especially on his mother's face. He cannot talk but makes sounds, which the mother can interpret; he shows dissatisfaction and laughs when he is happy.

Supplementary Table S1. Antiepileptic treatment profile

Year	Substance	Symptoms
2006 (1)	valproate (Depakine®)	Focal seizures of the arms and head with apnea
2006 (2)	valproate, levetiracetam (Keppra®)	Focal seizures of the arms and head with apnea
2007 (1)	oxcarbazepine (Trileptal®)	No seizures but hyponatremia
2007 (2)	zonisamide (Zonegran®)	No effect (seizures 5x / day)
2008	topiramate (Topamax®)	No seizures
2009	topiramate, phenytoin (Epilan-D®)	Tonic seizures with head deviation (left side) 4x / week
2010	topiramate, pregabalin (Lyrica®)	Seizures reduced to 1-2 / day
2013	topiramate, pregabalin, phenytoin, vigabatrin (Sabril®)	No Seizures but leucopenia
2015	topiramate, pregabalin, vigabatrin	Tonic seizures 3x / day
2018	rufinamide (Inovelon®), pregabalin, topiramate	Tonic seizures 5x / week
2019	pregabalin, lamotrigine (Gerolamic®), topiramate	No seizures

Supplementary Figure S2. Cerebral MRI (T2 sequence; at an age of 5 ½ years) revealing frontal and occipital cerebral atrophy. No focal temporal lesions.

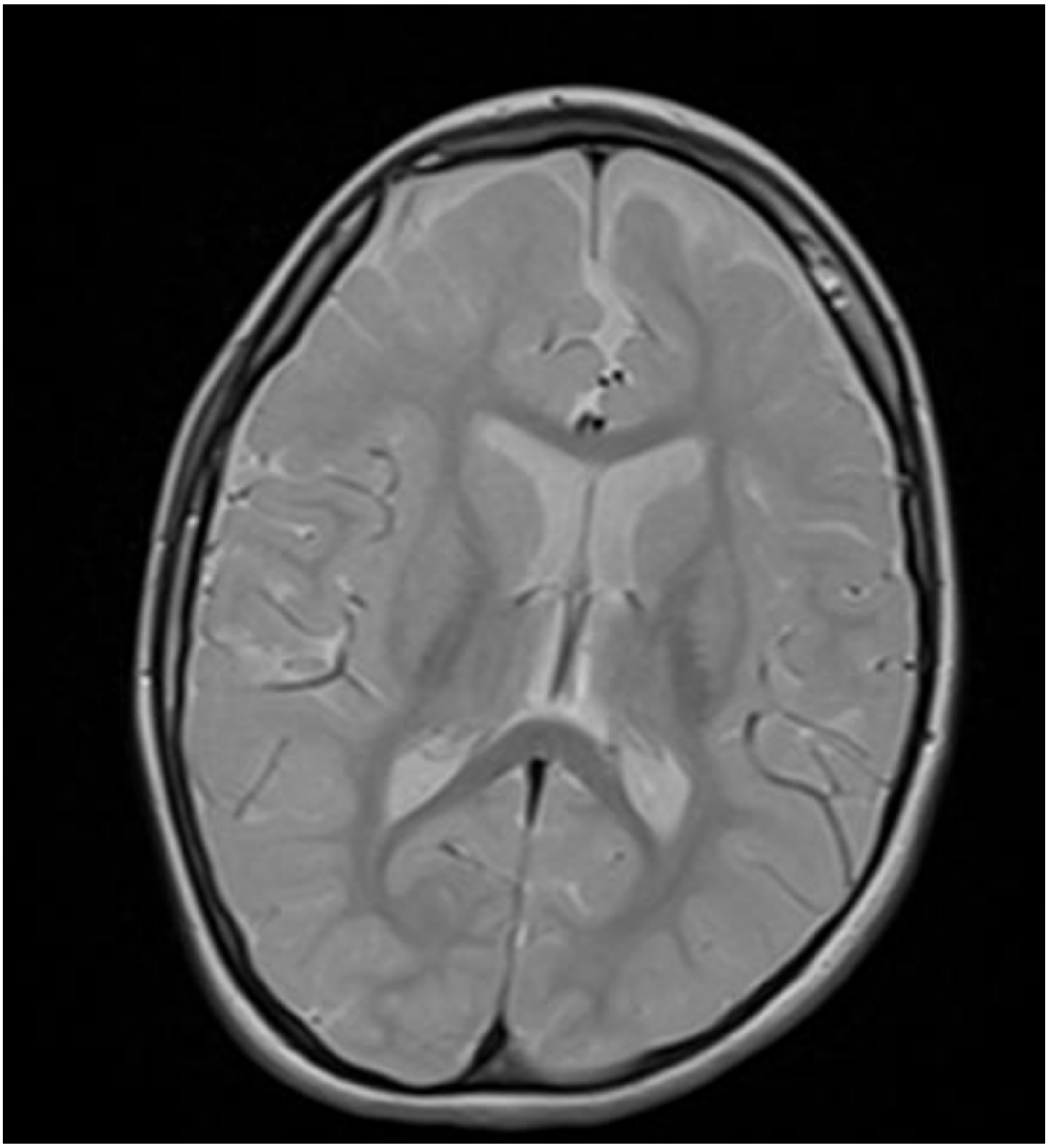

Supplementary Figure S3. (**a**) sleep EEG (age 4 years), interictal discharges in form of focal sharp waves and spike waves T3; (**b**) awake state EEG (age 8 years), background pattern, slow with theta and alpha waves with a frequency of 6-8/s, rhythmic delta waves, frontal beta-activity; otherwise no epileptic discharges (no clinical seizures).

a

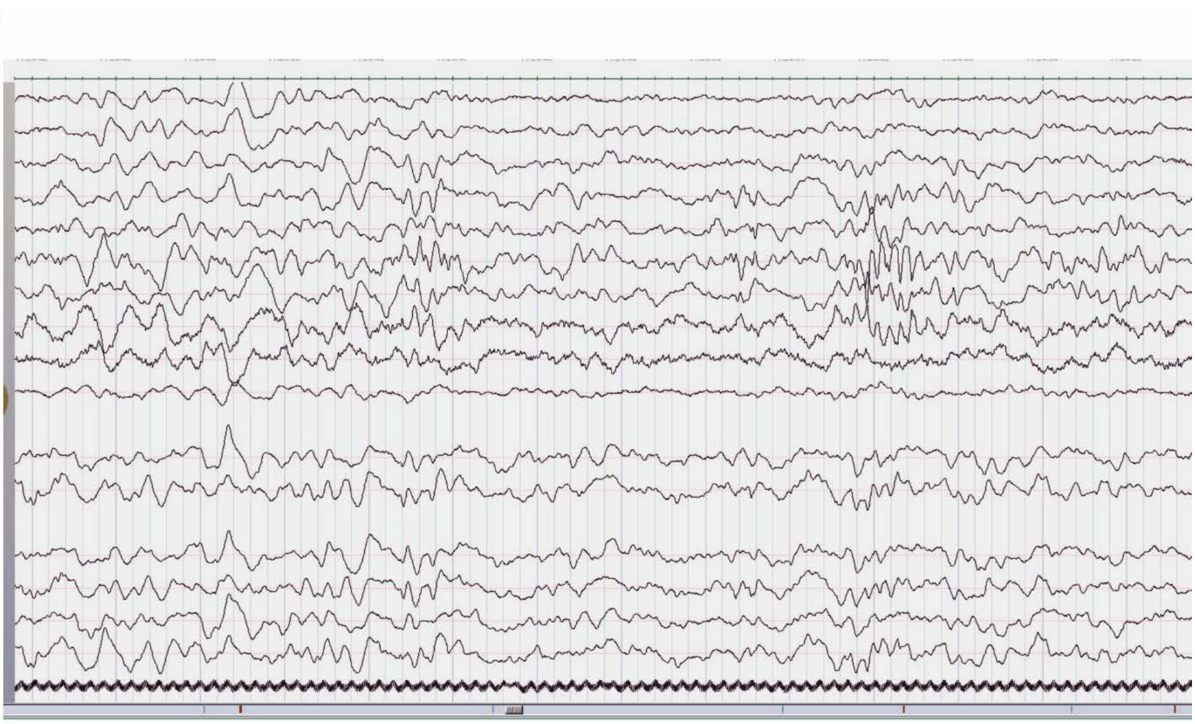

b

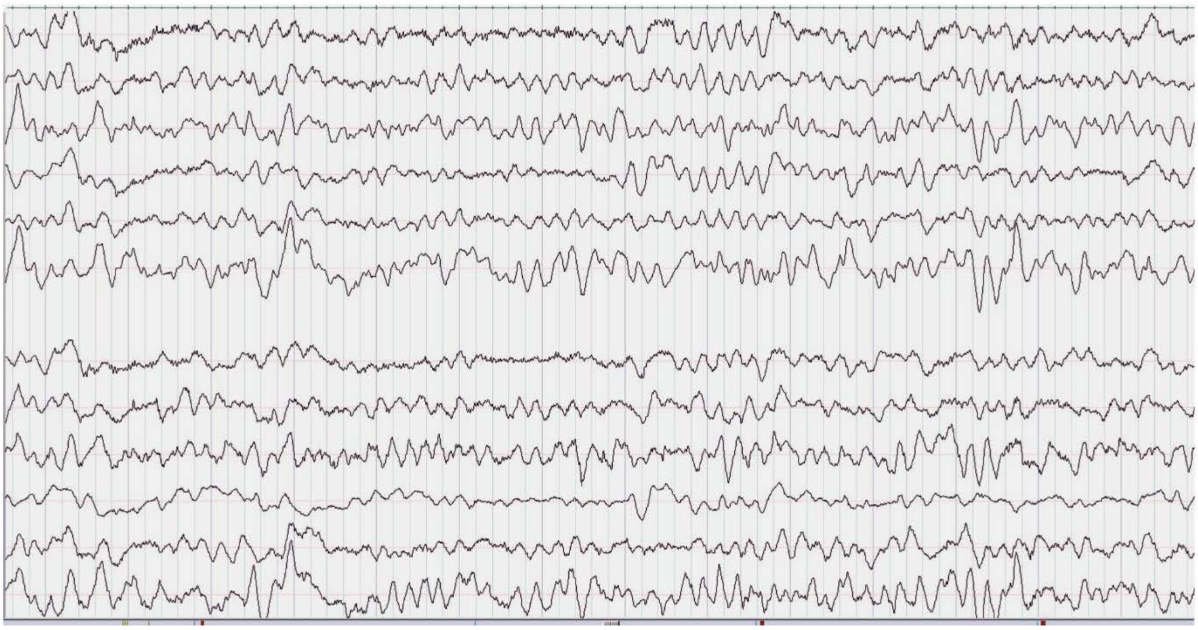

Supplementary Figure S4. Partial sequence alignment of all human $GABA_A$ receptor subunits. The A332 highlighted in red is conserved among most subunits, in comparison to the other two mutations in the TM3 which show conservation only among α subunits.

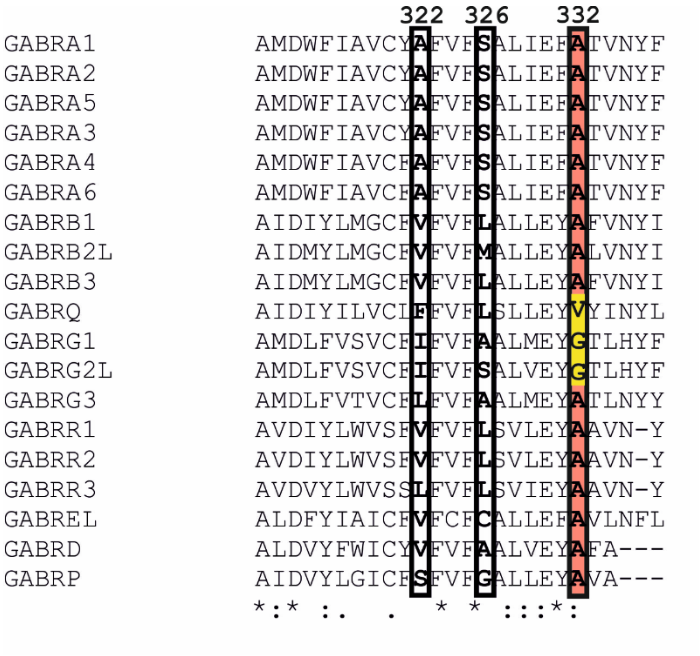

Supplementary Figure S5. Inhibition of binding of [^{3}H]-flunitrazepam to recombinant α1β3γ2 and α1A332Vβ3γ2 $GABA_A$ receptors. Data represent mean ± SEM derived from 6 individual experiments.

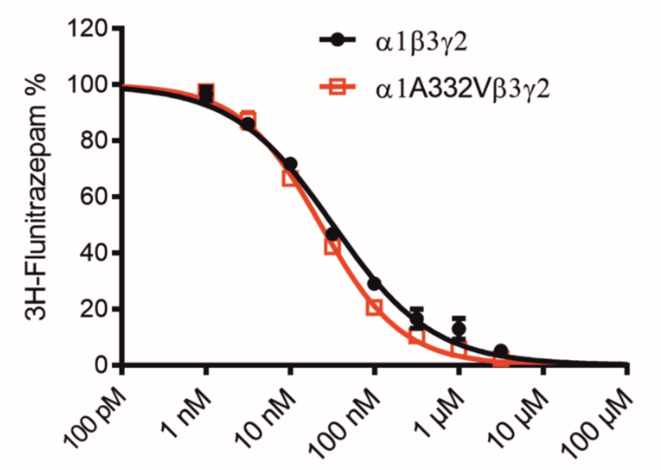

Supplementary Figure S6. Maximum current amplitudes elicited by 1 mM GABA. Bars and error bars represent mean ± SEM. Statistically significant differences were determined by two-tailed students *t*-test, where $p<0.05$; ***=$p<0.001$.

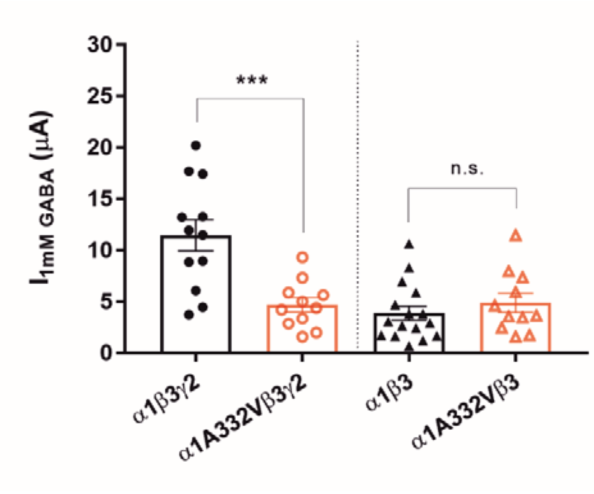

Supplementary Figure S7. (**a**) GABA dose response curve in α1β3 and α1A332Vβ3 taken from Figure 4a of the manuscript. Dashed lines indicate the levels of activation (% of Imax) used to analyse the extent of desensitization, namely 35% and 80%. The arrows mark the concentrations that were used for the subsequent analysis. (**b**) The extent of desensitization was determined at ~35% and ~80% activation in α1β3 and α1A332Vβ3. The mutated receptors desensitize significantly more than the wild-type receptors at comparable activation levels. (**c**) GABA dose response curve in α1β3γ2 and α1A332Vβ3γ2 taken from Figure 4b of the manuscript. Dashed lines indicate the levels of activation (% of Imax) used to analyse the extent of desensitization, namely 65%. The arrows mark the concentrations that correspond to the levels of activation used. (**d**) The extent of desensitization determined at ~65% activation in α1β3γ2 and α1A332Vβ3γ2. The mutated receptors desensitize significantly more than the wild-type receptors at comparable activation levels. Bars and error bars represent mean ± SEM. Statistically significant differences were determined by two-tailed students *t*-test, where $p<0.05$; **=$p<0.01$, ***=$p<0.001$.

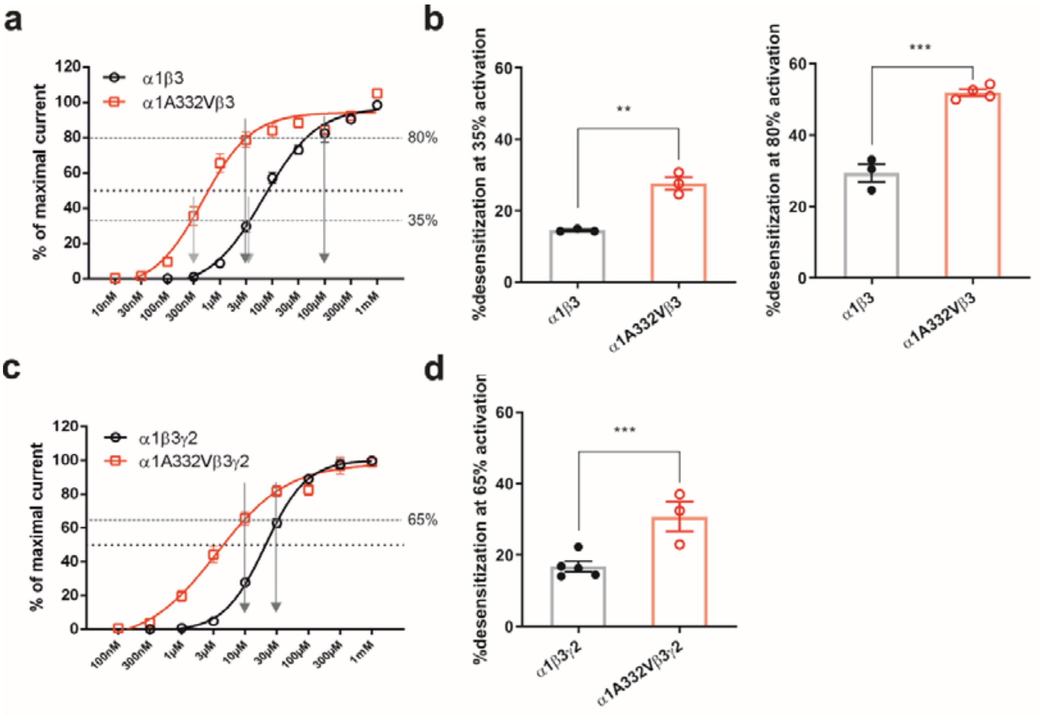

Supplementary Figure S8. Exemplary GABA dose response curves of α1β3 (**a**) and α1A332Vβ3 (**b**) with concentrations ranging from 1nM to 1mM GABA, and α1β3γ2 (**c**) and α1A332Vβ3γ2 (**d**) measured from 300nM to 1mM GABA.

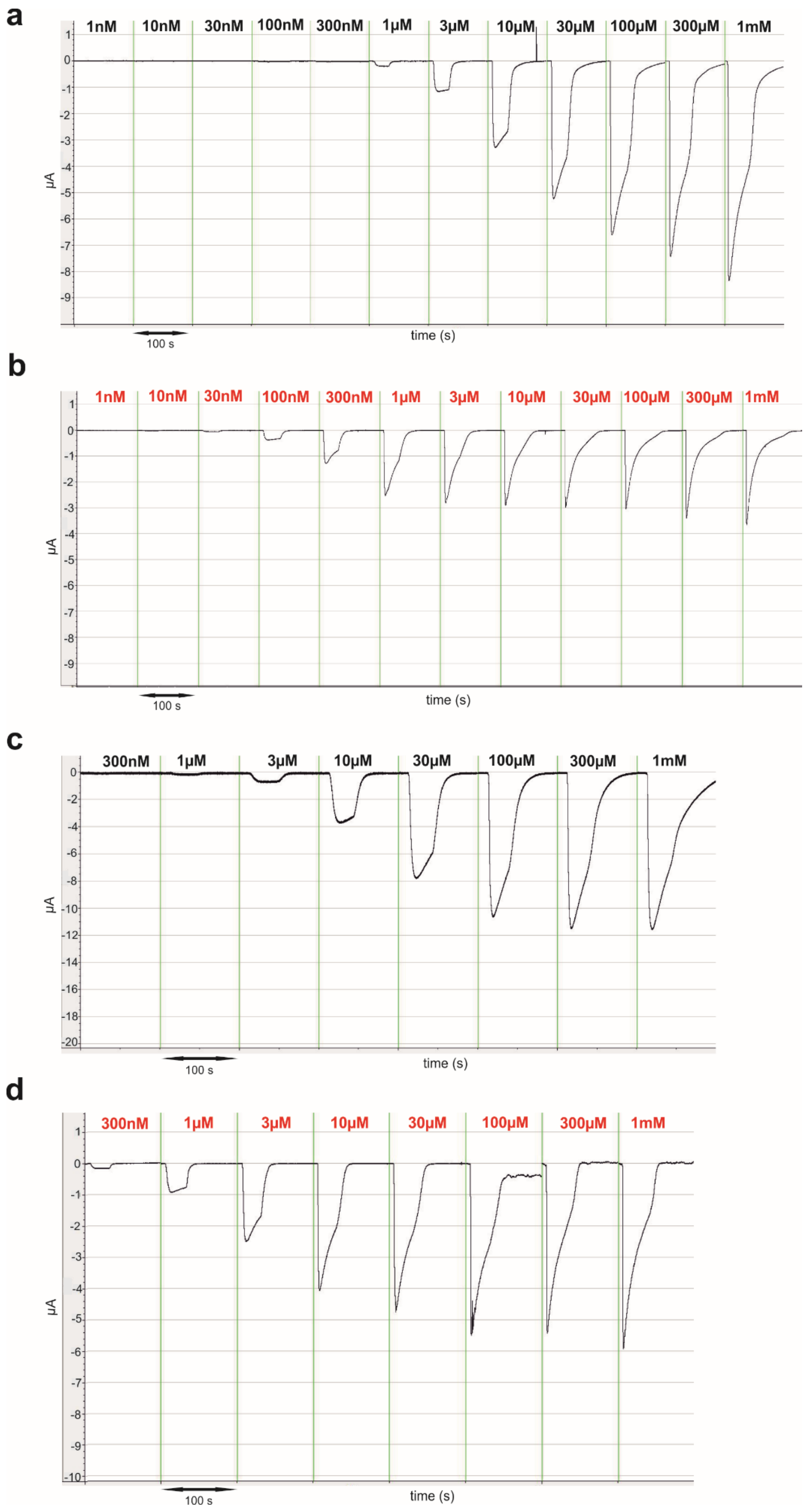

Supplement: Supplementary file 1 — Supplementary Information. [file 41598_2020_59323_MOESM1_ESM.pdf]
